# 2-Color Calcium Pump Reveals Closure of the Cytoplasmic Headpiece with Calcium Binding

**DOI:** 10.1371/journal.pone.0040369

**Published:** 2012-07-11

**Authors:** Zhanjia Hou, Zhihong Hu, Daniel J. Blackwell, Tyler D. Miller, David D. Thomas, Seth L. Robia

**Affiliations:** 1 Department of Cell and Molecular Physiology, Loyola University Chicago, Maywood, Illinois, United States of America; 2 Department of Biochemistry, Molecular Biology, and Biophysics, University of Minnesota, Minneapolis, Minnesota, United States of America; Cinvestav-IPN, Mexico

## Abstract

The sarco(endo)plasmic reticulum calcium ATPase (SERCA) undergoes conformational changes while transporting calcium, but the details of the domain motions are still unclear. The objective of the present study was to measure distances between the cytoplasmic domains of SERCA2a in order to reveal the magnitude and direction of conformational changes. Using fluorescence microscopy of live cells, we measured intramolecular fluorescence resonance energy transfer (FRET) from a donor fluorescent protein fused to the SERCA N-terminus to an acceptor fluorescent protein fused to either the N-, P-, or transmembrane domain. The “2-color” SERCA constructs were catalytically active as indicated by ATPase activity *in vitro* and Ca uptake in live cells. All constructs exhibited dynamic FRET changes in response to the pump ligands calcium and thapsigargin (Tg). These FRET changes were quantified as an index of SERCA conformational changes. Intramolecular FRET decreased with Tg for the two N-domain fusion sites (at residue 509 or 576), while the P- (residue 661) and TM-domain (C-terminus) fusions showed increased FRET with Tg. The magnitude of the Tg-dependent conformational change was not decreased by coexpression of phospholamban (PLB), nor did PLB slow the kinetics of Tg binding. FRET in ionophore-permeabilized cells was lower in EGTA than in saturating calcium for all constructs, indicating a decrease in domain separation distance with the structural transition from E2 (Ca-free) to E1 (Ca-bound). The data suggest closure of the cytoplasmic headpiece with Ca-binding. The present results provide insight into the structural dynamics of the Ca-ATPase. In addition, the 2-color SERCA constructs developed for this study may be useful for evaluating candidate small molecule regulators of Ca uptake activity.

## Introduction

The sarco(endo)plasmic reticulum calcium adenosine triphosphatase (SERCA) is the ion-motive ATPase responsible for maintaining the 7,000 fold Ca gradient [1] across the membrane of the endoplasmic reticulum. ER Ca stores are critical for cell signaling, and disordered SERCA function or regulation is pathogenic [2]. SERCA plays a particularly important role in striated muscle, where Ca release and reuptake determines the contraction and relaxation of the muscle. In the heart, dysfunctional Ca handling has been implicated in heart failure, so SERCA is of great interest as a potential therapeutic target.

**Figure 1:**
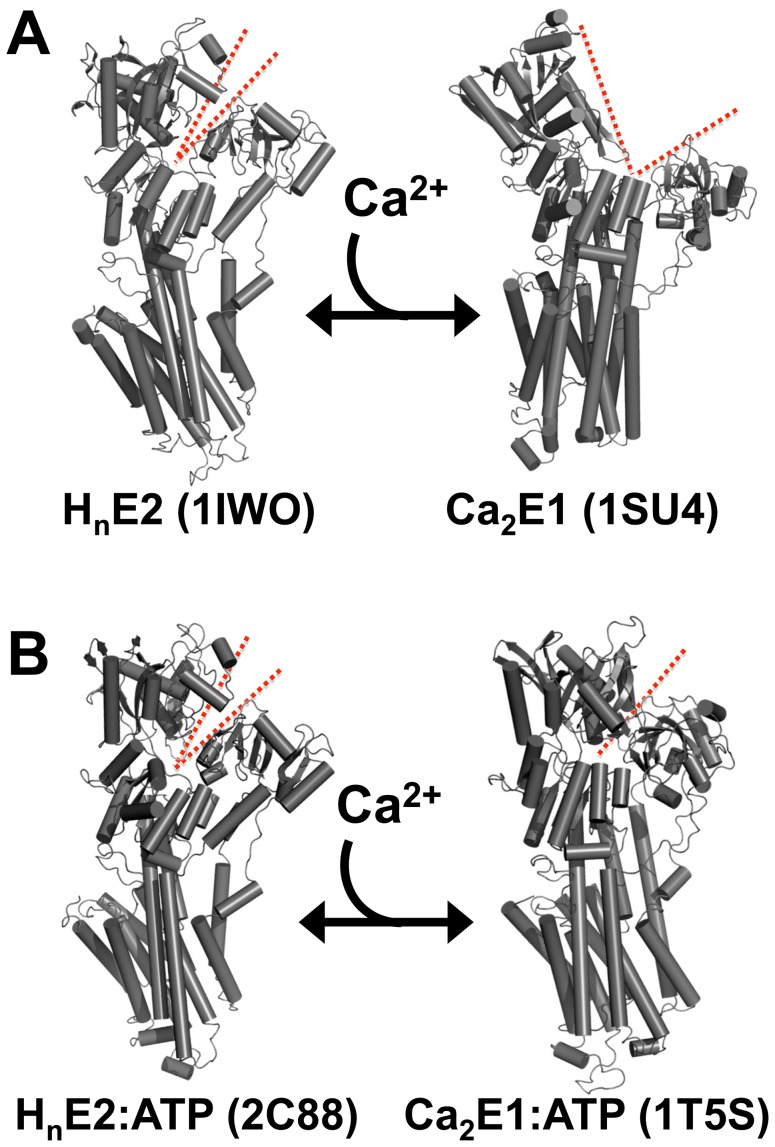
Possible conformational changes of SERCA in response to Ca binding. **A.** Opening of the cytoplasmic headpiece, increasing N- to A-domain distance. **B.** Closure of the cytoplasmic headpiece, decreasing domain separation. The cleft between the N- and A-domains is highlighted with dotted red lines.

The rational design of such therapies can benefit from an extensive literature on SERCA structure and function. SERCA is known to undergo conformational changes during catalytic cycling [3]. In particular, X-ray crystallography has provided many atomic resolution SERCA structures suggesting that the relative motions of SERCA cytoplasmic domains result in opening and closing of the cytoplasmic headpiece during the transition between the E1 (Ca-bound) and E2 (Ca-free) enzymatic substate (**Fig. 1**). Many aspects of these crystallographic results have been confirmed by other methods but some details of the domain motions are still unclear. In particular, the first two crystal structures of SERCA representative of E2 (**Fig. 1A**, 1IWO) [4] and E1 (**Fig. 1A**, 1SU4) [5] suggested that Ca-binding resulted in a large-amplitude (>20 Å) opening of the cytoplasmic headpiece. However, later crystal structures, determined in the presence of nucleotide analogs, suggest that the headpiece remains closed throughout the catalytic cycle. It has been hypothesized that the prevailing millimolar concentrations of nucleotide in live cells stabilize the headpiece in a perpetually closed state [6], [7]. Thus, the conformational changes may be smaller in magnitude than originally supposed [8]. Rather than opening with Ca, the headpiece may transition from a loosely closed E2 conformation (**Fig. 1B**, 2C88) [6] to a tightly closed conformation (**Fig. 1B**, 1T5S) [7]. A challenge for interpreting these high-resolution structures is that functionally important conformations may be overlooked by X-ray crystallography, which is biased toward compact conformations and well-ordered structures. Complementary alternative approaches are needed to address the magnitude and direction of changes in the architecture of the SERCA cytoplasmic headpiece.

To directly quantify SERCA headpiece conformational changes in live cells, we used a strategy of measuring intramolecular fluorescence resonance energy transfer (FRET) between fluorescent protein fusion tags directed to various sites on the SERCA cytoplasmic domains. The goal of these experiments was to determine how the domain separation distance changes with Ca-binding. This approach is similar to domain-domain FRET methods used in other studies [8], [9], [10], but offers the additional advantage of an intrasequence tag [11], [12], which can be fused to arbitrary sites on SERCA. In addition, the fluorescent construct is entirely biosynthetic and compatible with live cell measurements. By quantifying relative changes in probe separation distance we can gain insight into the conformational changes of the SERCA cytoplasmic headpiece.

## Methods

### Molecular Biology, Cell Culture, and Localization

**Figure 2:**
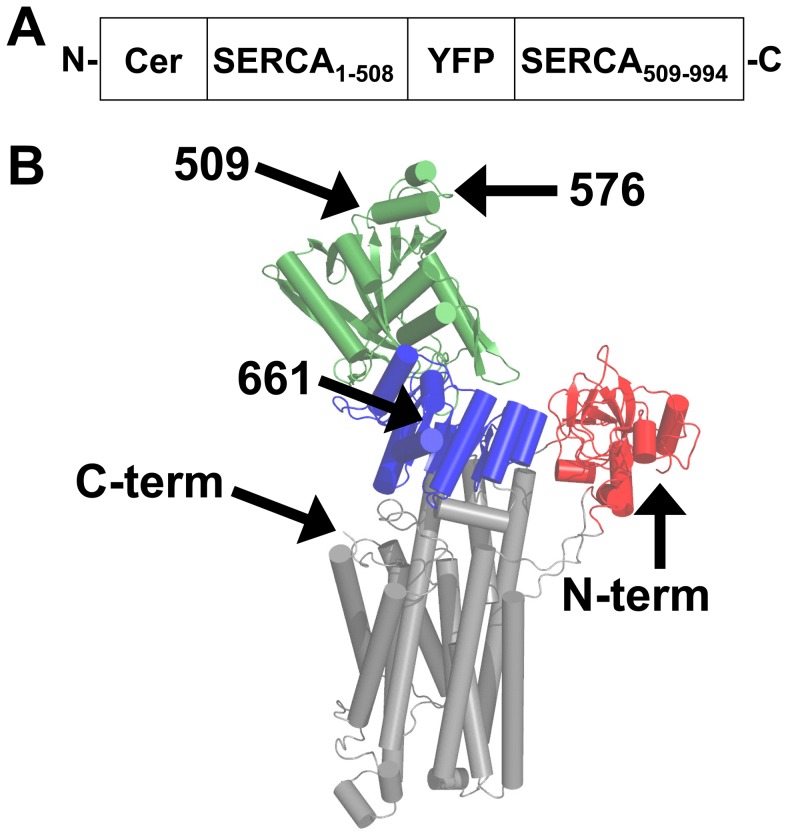
2-color SERCA constructs. **A.** Schematic diagram of construct 509. 2-color SERCA comprises SERCA2a with an N-terminal Cer and an intrasequence YFP. **B.** Fusion positions for Cer (N-term) and intrasequence YFP (509, 576, 661, or C-term).

mCerulean (Cer) was fused to the N-terminus of canine SERCA2a. This fusion position is in the A-domain of the pump, highlighted in red in **Fig. 2**. YFP was fused as an intrasequence tag before residue 509 or 576 in the N-domain (**Fig. 2**B, green), or 661 in the P-domain (**Fig. 2**B, blue) or at the SERCA C-terminus in the TM domain (**Fig. 2**B, grey). Structures provided in **Fig. 1** and **Fig. 2** correspond to the isoform SERCA1a, which is 84% homologous to SERCA2a. Intrasequence fusion positions were chosen using E1/E2 x-ray crystal structures of SERCA1a to identify unstructured loops and predict large relative distance changes from E1 to E2 [4], [5]. The other major design goal was to avoid regions important for SERCA function [13]. Fluorescence microscopy of AAV-293 cells (Agilent, Santa Clara, CA) expressing 2-color SERCA was performed in glass-bottom chambered coverslips (Matek Corporation, Ashland, MA) coated with poly-D-lysine (Sigma-Aldrich) 18–24 hours post-transfection with MBS mammalian transfection kit (Stratagene, La-Jolla, CA). To determine the effect of Ca on SERCA structure, cells were permeabilized for 1 minute in 50 µM ionomycin in growth medium (90% Dulbecco’s Modified Eagle Medium, 10% fetal bovine serum, 1% glutamine). To reduce intracellular Ca, cells were permeabilized with 50 µM ionomycin in 5 mM EGTA in phosphate-buffered saline or relaxing solution (100 mM KCl, 5 mM EGTA, 5 mM MgCl_2_, 3.2 mM ATP, 10 mM imidazole pH = 7.2). Confocal microscopy was performed using a Leica SP5 confocal microscope equipped with a 1.3 N.A. 63 X water immersion objective. Excitation was accomplished with Ar laser illumination at 458 nm for Cer, 514 nm for YFP and FM 4-64, with emission bands of 467–512 nm for Cer, 528–565 for YFP, and 674–797 nm for FM 4–64, using sequential image acquisition. Total internal reflection fluorescence (TIRF) microscopy was performed with a Nikon inverted microscope (Ti-e) equipped with a 100X oil-immersion objective with (N.A. = 1.49) and a cooled CCD camera (Coolsnap K4, Photometrics, Tucson, AZ). Through-objective TIRF excitation was achieved with a 449 nm diode laser (for Cer) or 514 nm Ar laser (for YFP). The laser incident angle was adjusted to create an evanescent field that illuminated the plasma membrane in contact with the surface substrate and a thin section of endoplasmic reticulum. Widefield lamp excitation was used for FRET measurements, using computer-controlled filter wheels, as previously described [14], [15], [16], [17], [18], [19]. Fluorescent images were recorded with a cooled EM-CCD camera (Andor Ixon, Belfast, Northern Ireland).

### 2-color SERCA Enzymatic Activity

To evaluate Ca transport activity for 2-color SERCA constructs we performed a live-cell Ca uptake assay, as previously described [14]. Heterogeneously transfected populations of AAV-293 cells were incubated with cell permeant Ca indicator dye X-rhod 1 (AM) (Invitrogen, Carlsbad, CA). Transfected and untransfected cells were distinguished on the basis of the intensity of YFP fluorescence emission. Release of Ca from intracellular stores was accomplished by stimulating purinergic receptors with extracellular application of 100 µM ATP. Accumulation of Ca in the cytosol was quantified as an increase in X-rhod 1 fluorescence, and was the net result of Ca release counterbalanced by Ca extrusion and uptake processes, including SERCA activity. Exogenous SERCA activity was detected as a decrease in ATP-stimulated cytosolic Ca accumulation relative to untransfected cells in the same microscopic field. 3 minutes after application of extracellular ATP, cells were treated with 10 µM Tg to determine the size of the Ca store remaining in the ER. The magnitudes of the ATP- and Tg-dependent Ca transients were quantified by integrating the area under the trace of X-rhod 1 fluorescence vs. time. The integrated area of Ca transients in 2-color SERCA transfected cells was compared to corresponding control (untransfected cells) using a paired t-test, with a p-value of <0.05 taken to indicate a significant difference. To determine whether the 2-color SERCA samples were regulated by phospholamban (PLB), cells transfected with 2-color SERCA were compared to separate samples transfected with both 2-color SERCA and YFP-PLB using an unpaired t-test, with a p-value of <0.05 taken to be a significant difference. For Cer-SERCA2a and construct 509, enzymatic activity was also quantified in cell homogenates by spectrophotometric measurement of the rate of NADH consumption in an enzyme-coupled activity assay, as previously described [20]. Conventional cell transfection did not yield adequate expression of SERCA protein for this assay, so AAV-293 cells were infected with adenoviruses encoding Cer-SERCA2a or 509, resulting in much greater protein expression [21], as quantified by immunoblotting [22].

### FRET Measurement

FRET was quantified as previously described [14], [15], [17] using the E-FRET (3-cube) method [23]. FRET efficiency (E) was calculated according to the relationship
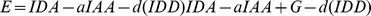
where I_DD_ is the intensity of fluorescence emission detected in the donor channel (472/30 nm) with excitation of 427/10 nm; I_AA_ is acceptor channel (542/27 nm) emission with excitation of 504/12 nm; I_DA_ is the “FRET” channel, with 542/27 nm emission and excitation of 427/10 nm; *a* and *d* are cross-talk coefficients determined from acceptor-only or donor-only samples, respectively. We obtained values of *d* = 0.7 (for Cer) and *a* = 0.074 (for YFP). *G* is the ratio of the sensitized emission to the corresponding amount of donor recovery, which was 3.2 for this setup. Probe separation distance (*R*) was calculated from the relationship described by Förster, *R* = (*R*_0_)[(1/*E*)−1)^1/6^, where *E* is the measured FRET value and *R*_0_ is the Förster radius, which is 49.8 Å for the Cer-YFP pair [24]. E-FRET measurements were also compared with FRET obtained by the photobleaching method [16], [18], [19]. All error bars represent mean ± SE.

## Results

### Activity of 2-color SERCA

**Figure 3:**
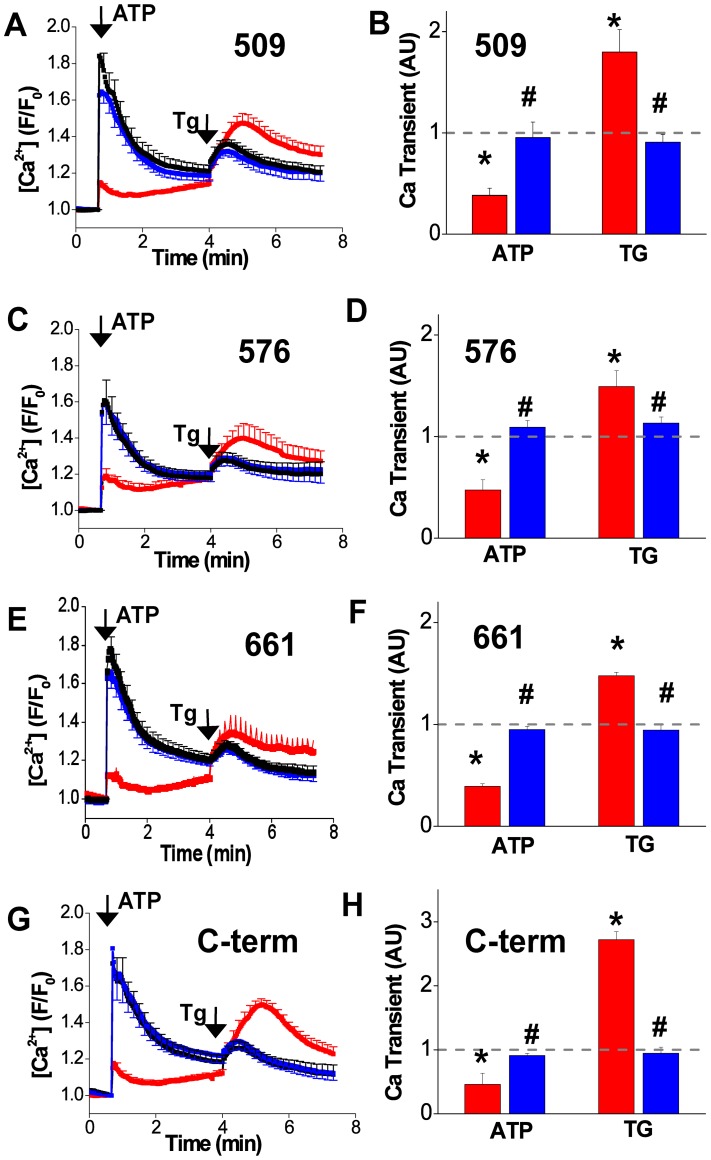
Ca uptake in live cells quantified from changes in cytosolic Ca measured by X-rhod 1 fluorescence. Application of extracellular ATP elicited an increase in cytosolic Ca in untransfected cells (black). This Ca transient was largely abolished in cells in the same microscopic field that expressed 2-color SERCA constructs (red), suggesting Ca uptake by 2-color SERCA. Coexpression of PLB partially restored the ATP-releasable Ca transient, consistent with inhibition of 2-color SERCA by PLB. The Ca content of the ER was evaluated by application of 10 µM Tg. Cells expressing 2-color SERCA (red) had a larger Tg-releasable ER content compared to untransfected cells (black). This difference was abolished by coexpression of PLB (blue). **A.** Uptake of Ca by 509. **B.** Summary of 509 uptake (normalized area under the peak). **C.** 576. **D.** Summary of 576. **E.** 661. **F.** Summary of 661. **G.** C-term. **H.** Summary of C-term. * indicates 2-color SERCA was significantly different from paired control (untransfected cells). # indicates 2-color SERCA + PLB was significantly different from unpaired 2-color SERCA alone.

To verify that the fluorescent protein fusion tags did not disrupt SERCA catalytic function, we measured Ca uptake for all constructs using a live-cell Ca uptake assay [14]. **Fig. 3** shows that untransfected cells responded to application of extracellular ATP (arrow “ATP”) with a large cytosolic Ca transient that was detected as an increase in the fluorescence of the Ca-sensitive dye X-rhod-1 (**Fig. 3**, black traces). This accumulation of Ca in the cytosol was almost completely prevented in cells transfected with 2-color SERCA (**Fig. 3**, red traces), suggesting that the transport activity of exogenous SERCA counterbalanced Ca release. In this experiment, heterogeneous expression levels in a population of AAV-293 cells permit comparison of transfected and untransfected cells in the same field. Notably, Tg-releasable ER Ca content was greater in cells transfected with 2-color SERCA (**Fig. 3**, red, “Tg”). Co-transfection of 2-color SERCA constructs with YFP-PLB partially restored the observed Ca transient and reduced the Tg-releasable ER Ca content (**Fig. 3**, blue), consistent with inhibition of SERCA by PLB. For all constructs, the area under the curve was quantified for ATP- and Tg-dependent Ca transients and summarized in **Fig. 3**B, D, F, H as normalized values relative to untransfected cells.

**Figure 4:**
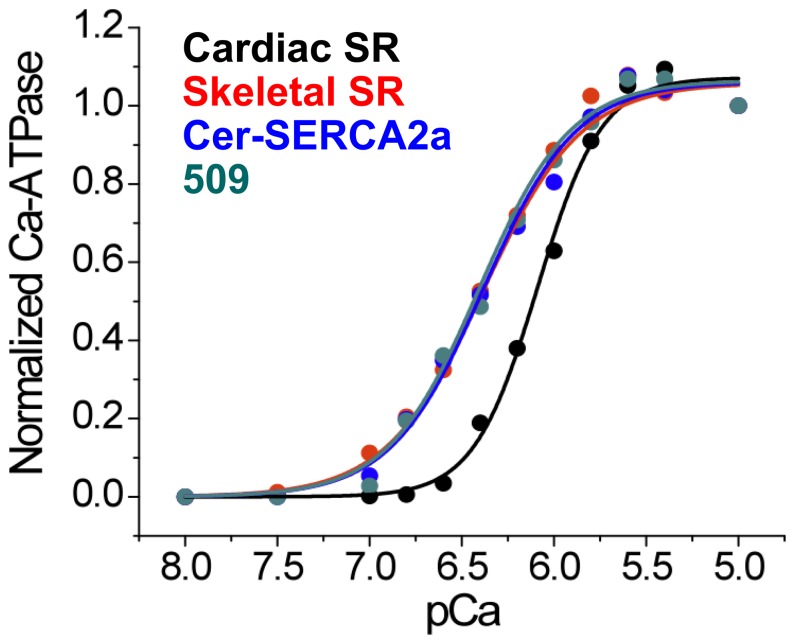
Calcium-dependent ATPase activity of Cer-SERCA2a and 2-color SERCA (construct 509) measured in cell homogenates. The Ca-sensitivity was indistinguishable from that of skeletal SR (red) and greater than that of cardiac SR (black), presumably due to the presence of phospholamban in the latter. The specific ATPase activity (ATP hydrolyzed per s per SERCA2a at 37°C) at saturating Ca^2+^ (pCa 5.0) was indistinguishable for cardiac SR, Cer-SERCA2a, and 2-color SERCA.

Pump activity was also evaluated for some constructs using an enzyme-linked ATPase assay performed on cell homogenates. **Fig. 4** shows a comparison of the Ca-dependent ATPase activity of cardiac SR (black) and skeletal SR (red) controls versus homogenates of AAV-293 cells infected with adenoviruses encoding Cer-SERCA2a (blue) [14] or 2-color SERCA construct 509 (cyan) after subtraction of endogenous ATPase activity. 2-color SERCA yielded a pKCa of 6.4 and a Hill coefficient of 1.7, which is in agreement with skeletal light SR control (red) (**Fig. 4**). ATPase specific activity was determined to be 3.79±0.98 s^−1^ per SERCA for Cer-SERCA2a and 4.98±1.68 s^−1^ per SERCA for construct 509. These values compare favorably that of porcine cardiac SR, which yielded a specific activity of 3.08±0.18 s^−1^ per SERCA. Overall, the data indicate that the fluorescent protein fusion tags are benign for SERCA catalytic function and regulation.

### 2-color SERCA Localization and FRET

**Figure 5:**
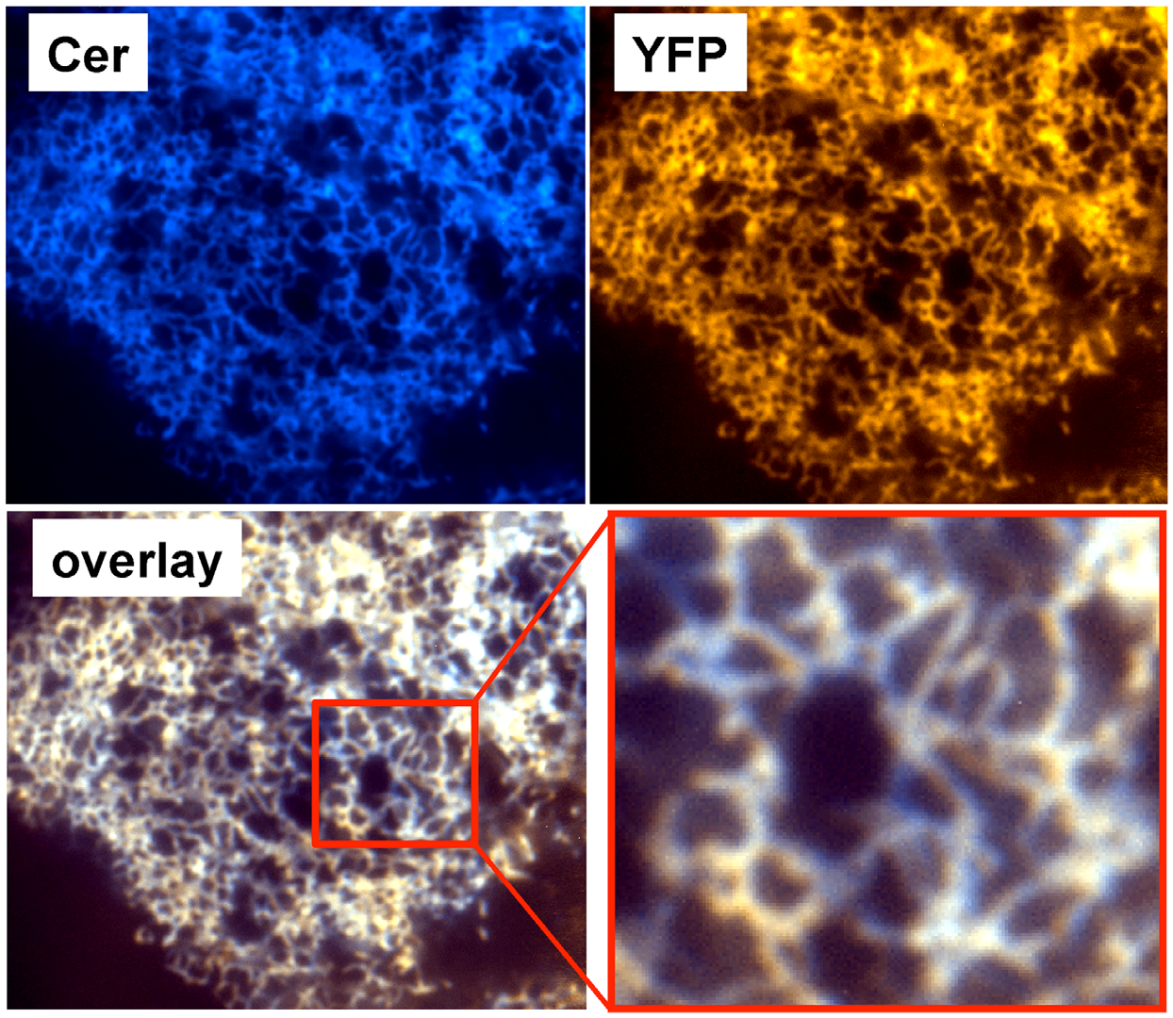
TIRF microscopy of AAV-293 cells expressing 2-color SERCA (509). Fluorescence was distributed in a reticulated pattern consistent with ER localization.

**Figure 6:**
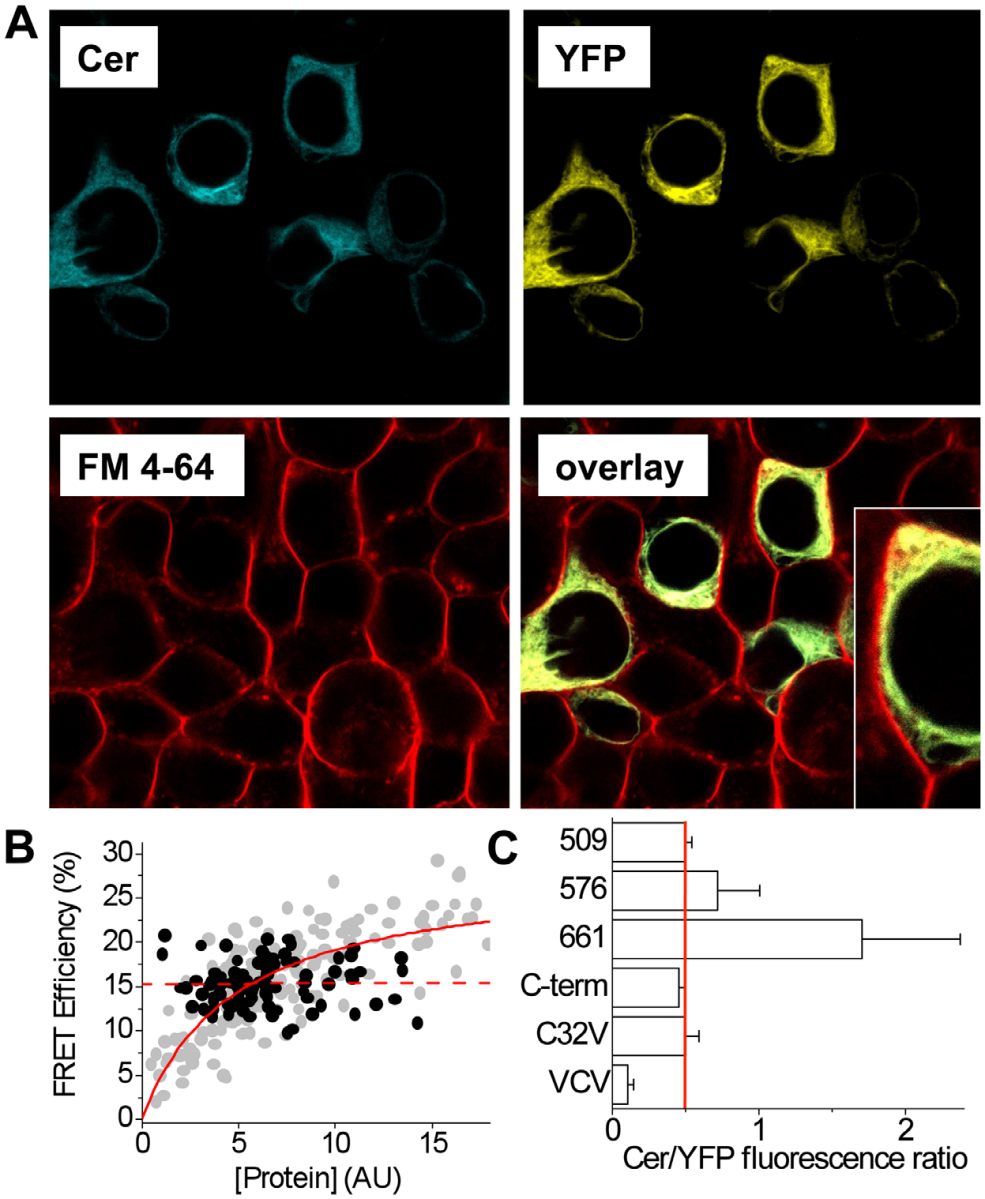
Characterization of 2-color SERCA. **A.** Confocal microscopy showed 2-color SERCA was localized in internal perinuclear membranes. Plasma membranes were counterstained with FM 4-64. Inset shows an enlarged view. **B.** 2-color SERCA showed concentration-independent intramolecular FRET (black points, dotted line). A hyperbolic dependence on protein expression was observed for intermolecular FRET from Cer-SERCA to YFP-PLB (grey points, solid line). **C.** The brightness ratio Cer/YFP sensitivity ratio for microscopy setup was 0.5 (red line). Deviation from this value suggests a Cer/YFP stoichiometry other than 1∶1. C32V and VCV are controls with 1∶1 and 1∶2 stoichiometry, respectively (*25*).

All of the constructs showed fluorescence localization patterns consistent with localization in the endoplasmic reticulum. TIRF microscopy of cells expressing construct 509 showed that Cer and YFP signals were highly colocalized in a reticulated pattern (**Fig. 5**). **Fig. 6**A shows confocal microscope images obtained by sequential acquisition of signals from Cer, YFP, and the cell-impermeant membrane dye FM 4–64. The red fluorescence of FM 4–64 is localized to the plasma membrane, while the Cer and YFP fluorescence of 2-color SERCA (509) is localized to internal, perinuclear membranes.

For this construct we observed >15% FRET efficiency that was not dependent on protein expression level (**Fig. 6**B, black points and dotted line). This concentration independence of intramolecular FRET is in contrast to our previous observations of intermolecular FRET. For example, FRET from SERCA to PLB showed a hyperbolic concentration dependence, both in previous experiments [14], [15], [16], [17], [18] and in the present study (**Fig. 6**B, grey points and solid line). The lack of concentration dependence suggests that the FRET observed for 2-color SERCA is not due to protein-protein interactions, such as SERCA dimerization [25], and that nonspecific FRET [15] is not a significant factor for these experiments.

The observed Ca transport and ATPase activity (**Fig. 3**, **Fig. 4**) suggest that the sections of protein derived from SERCA were folded into the correct conformation. To test whether the donor and acceptor fusion tags were properly folded, we compared the fluorescence of Cer and YFP for all constructs. We have previously determined the illumination and detection configuration used in the present study yields a Cer/YFP brightness ratio of 0.5 [14], [15], [17]. **Fig. 6**C shows that the control construct C32V yielded a Cer/YFP ratio of 0.5, consistent with a 1∶1 stoichiometry of Cerulean and Venus fluorescent proteins [26]. Another control composed of a Cer fused to 2 Venus yielded a much lower Cer/YFP ratio consistent with a 1∶2 stoichiometry and significant quenching of the Cer protein by FRET. Two of the 2-color SERCA constructs gave the expected Cer/YFP ratio of approximately 0.5, suggesting correct maturation of the fluorescent protein tags with 1∶1 stoichiometry (**Fig. 6**C, 509 and C-term). The other two constructs showed increased Cer/YFP ratios and a larger cell-to-cell standard deviation (**Fig. 6**C, 576 and 661), suggesting that the intrasequence YFP tag was not achieving uniformly correct folding or maturation. Incomplete fusion tag maturation is expected to reduce the measured FRET and decrease the dynamic response for these constructs. The apparent misfolding of 576 and 661 intrasequence YFP was not improved by reducing the cell culture incubation temperature from 37°C to 25°C (not shown).

### SERCA Conformational Changes

**Figure 7:**
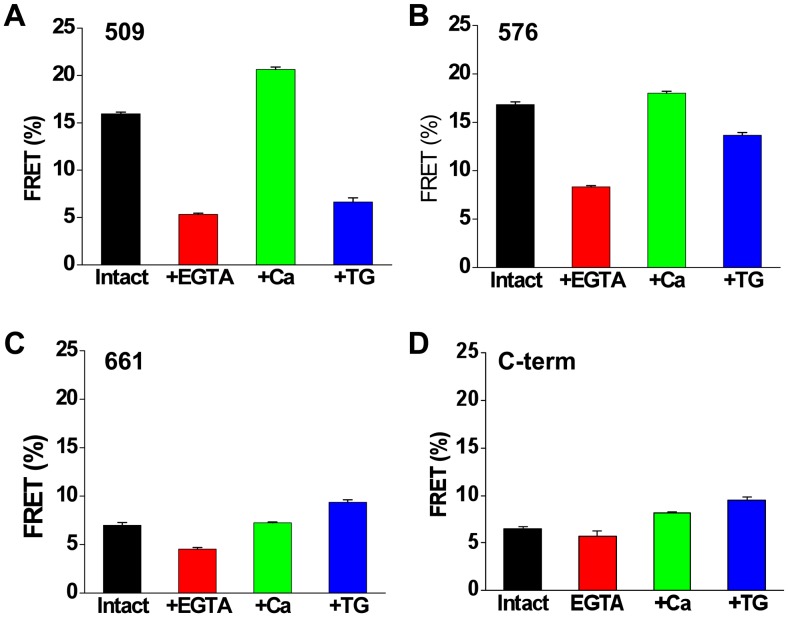
Dynamic FRET changes of SERCA constructs expressed in AAV-293 cells. Data represent control intact cells (black), cells permeabilized with ionophore with 5 mM EGTA (red) or 2 mM Ca (green) in the extracellular solution, or intact cells treated with thapsigargin (blue).

The average FRET observed for 2-color SERCA was found to depend on the YFP insertion site and the enzymatic substate of the pump (**Fig. 7**). For control unpermeabilized cells, the highest FRET was observed for constructs 509 (**Fig. 7**A) and 576 (**Fig. 7**B) (>15%). These constructs have the YFP acceptor inserted in the top of the N-domain (**Fig. 2**B). The P-domain insertion site (661) and the C-terminal acceptor position gave the lowest initial FRET. Ionophore permeabilization in the presence of 5 mM extracellular EGTA caused a decrease in FRET for all constructs (**Fig. 7**, red) over approximately 50 minutes. Treatment with ionophore in high extracellular Ca resulted in increased FRET (**Fig. 7**, green) in several minutes. While the magnitude of the change with Ca varied with YFP insertion site (**Fig. 7**), the direction of the Ca-dependent FRET change was positive for all constructs, suggesting that the conformational change from E2 to E1 decreased the distance between the donor and acceptor. This is in contrast to the response of 2-color SERCA constructs to Tg, which varied in magnitude and direction depending on the YFP insertion site (**Fig. 7**, blue).

**Figure 8:**
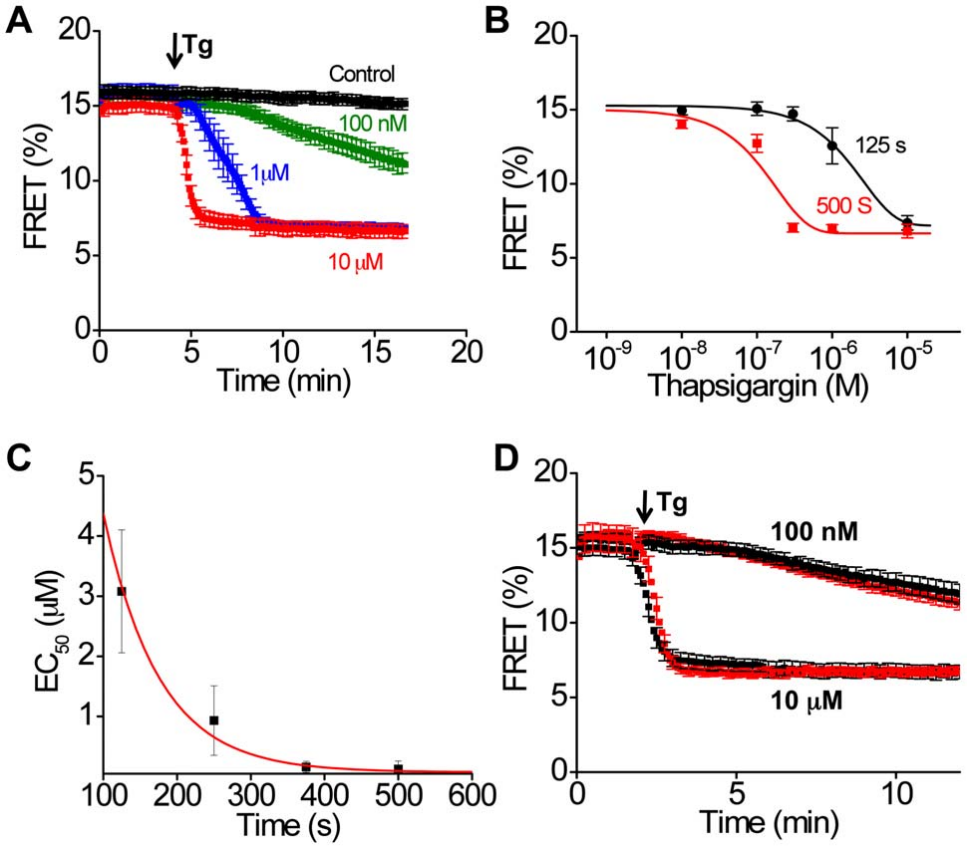
Tg-dependent conformational changes of 2-color SERCA (construct 509). **A.** The rate of change of FRET was Tg concentration dependent. **B.** Tg titration of 2-color SERCA, measured at different time points. **C.** The time dependent shift in apparent affinity of SERCA for Tg. **D.** The rate of Tg binding was not changed by coexpression of PLB with 2-color SERCA (red) compared to 2-color SERCA alone (black).

**Figure 9:**
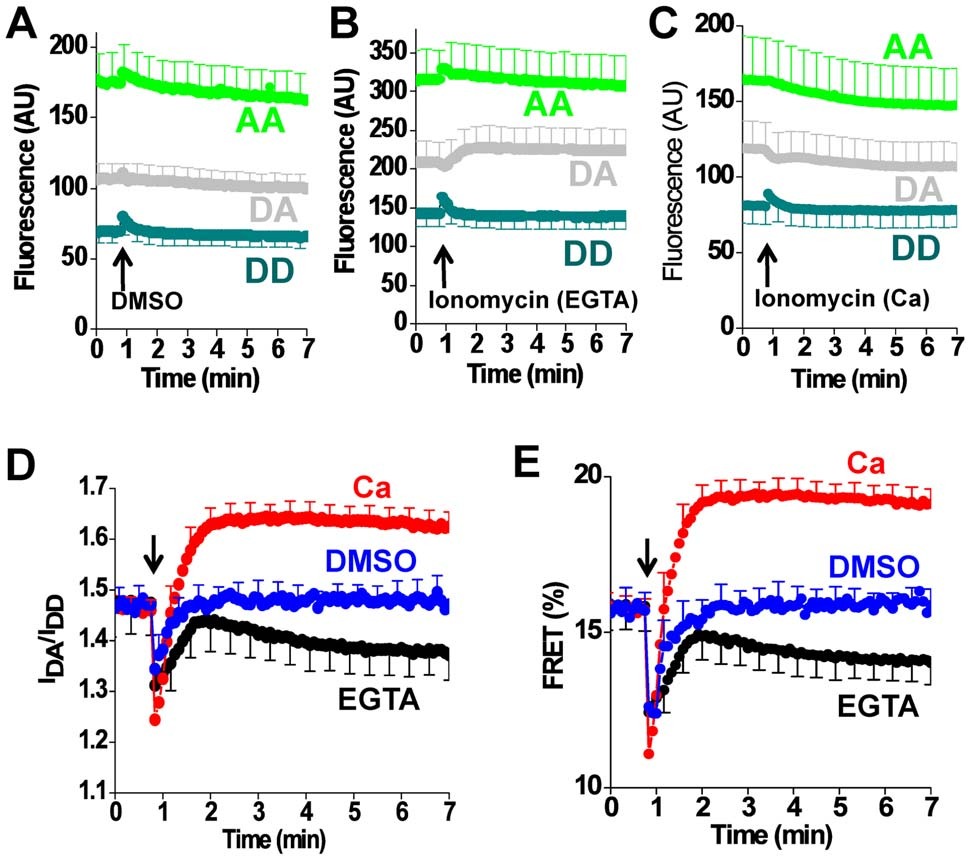
Ca^2+^-dependent conformational changes of 2-color SERCA (construct 509). **A.** Fluorescence emission measured in the donor channel (DD) and acceptor channel (AA), and the acceptor emission with donor excitation (DA) after the addition of DMSO vehicle. **B.** Fluorescence emission after addition of ionomycin in 2 mM Ca. **C.** Fluorescence emission after addition of ionomycin in the presence of EGTA. **D.** The ratio of DA and DD fluorescence intensities suggest that FRET is increased with Ca (red), and decreased with EGTA (black) compared to DMSO vehicle (blue). **E.** The corresponding FRET efficiency calculated according to the E-FRET method.

Additional characterization of construct 509 showed that the time course of the Tg-dependent FRET change was dependent on the concentration of Tg applied to the cells (**Fig. 8**A), with concentrations <1 µM requiring tens of minutes to produce a full effect. One consequence of the slow FRET response at low concentrations of Tg is that the apparent EC_50_ decreased over time. This is evident in **Fig. 8**B as a left shift in the binding curve with time. This change is quantified in **Fig. 8**C. The apparent upper limit estimate of EC_50_ (obtained from the final EC_50_ value) was approximately 100 nM, which is compatible with the subnanomolar affinity of the Tg-SERCA complex [27]. Notably, the timecourse of Tg binding was not significantly affected by coexpression of excess PLB, regardless of Tg concentration (**Fig. 8**D). These data are not consistent with the proposal that PLB protects SERCA from Tg by stabilizing a conformation that is not receptive to Tg [28]. The timecourse of the Ca dependent FRET change also showed slow kinetics. **Fig. 9** shows the response of construct 509 to addition of DMSO (**Fig. 9A** **)** vehicle or ionomycin to cells in the presence of Ca (**Fig. 8B**) or in EGTA (**Fig. 8C**). The ratio of the DA/DD signals is shown in **Fig. 8D**, and the corresponding FRET efficiency is given in **Fig. 9E**. The initial negative deflection (at arrow) in **Fig. 9D** and **Fig. 9E** is an optical artifact that was transient in duration. We attribute this to the mixing that occurs with addition of a large volume of solution to the bath surrounding the cells. Note that the rate of Ca flux is limited by the slow transport of Ca by ionomycin, and this limitation is most significant for EGTA experiments in which micromolar intracellular Ca is dialyzed against nanomolar extracellular Ca. Data from experiments such as **Fig. 8A** or **Fig. 9E** are summarized in **Fig. 7**, using the final FRET value obtained after equilibrium was achieved.

## Discussion

### Domain Separation Distance Change with Ca-binding

The principal finding of this study is that SERCA intramolecular FRET is increased by Ca binding for all four 2-color SERCA constructs. This suggests a decrease in the distance between the Cer donor fluorophore and the YFP acceptor, which reveals that the SERCA cytoplasmic headpiece becomes more compact after Ca binds to the SERCA transmembrane domain. The apparent donor-acceptor separation distances are summarized in Table 1. Several uncertainties apply to these values. Distances were calculated using the assumption that the average relative dipolar orientation of the probes was random (κ^2^ = 2/3). Nonspecific FRET was not subtracted from the measured FRET values, because there was no evidence of a concentration-dependent increase in FRET (**Fig. 6**B). The position of the fluorescent chromophores is not known precisely, as they are attached with flexible 2-residue linkers to SERCA cytoplasmic domains. Finally, the FRET efficiency measured in a steady-state experiment represents an average value that may integrate the contributions of multiple structural subpopulations. All of these unknown parameters complicate comparison of absolute distance measurements with X-ray crystal structures. However, assuming that the uncontrolled factors are not significantly different for E1 and E2, we analyzed the relative change in FRET to gain insight into SERCA conformational changes. Compared to EGTA, the apparent probe separation distance decreased with high Ca by approximately 23% (from 80 Å to 62 Å) for the most responsive construct, 509 (Table 1). This change was somewhat smaller in magnitude than the 30+ Å change predicted by the first X-ray crystallographic structures (**Fig. 1**A), but larger than that measured by other FRET studies [8], [9], [29], Most notably, the distance change observed here was opposite in direction compared to early crystal structure predictions [4]. The apparent decrease in FRET distance with Ca also contrasts with previous FRET studies that used reactive dyes as donors/acceptors. This may be due to differences in labeling strategies. FRET pairs on the N- and A- domains are expected to be maximally sensitive to SERCA headpiece conformational changes, and are benign for pump function (**Fig. 4**). In contrast, some dye conjugation chemistries result in inactivation of SERCA catalytic activity [8], [29]. The Ca-dependent decrease in probe separation distance observed here suggests closure of the cytoplasmic headpiece (**Fig. 1**B), and this was observed for all of the 2-color SERCA constructs (Table 1). The data are compatible with recent crystallographic studies, which collectively suggest the tightest closure of the cytoplasmic headpiece is in Ca-bound (E1) substates. Specifically, if the nucleotide-free Ca_2_E1 structure [5] is excluded, a comparison of the major E1 [7], [30], [31] and E2 [4], [6], [30], [31], [32], [33] crystal structures shows that the average separation distance between the N-terminal Cer fusion site (before residue 1) and the intrasequence YFP insertion site before residue 509 decreases with Ca binding. However, the large scale of the FRET distance change was somewhat surprising, as the nucleotide-bound states observed by X-ray crystallography all have rather closed structures (Fig. 1B), leaving little room for large-amplitude translation of the A- and N-domains. It is possible that open E2 conformations have been overlooked because they are relatively disordered, without domain-domain contacts to stabilize them for crystallization. Supporting the hypothesis of large-amplitude headpiece transitions is a recent study using fast scanning atomic force microscopy, which demonstrated 23 Å changes in the height of the SERCA headpiece relative to the surface of the bilayer during catalytic cycling [34]. Our data are also consistent with molecular dynamics simulations [35] performed on SERCA starting at the open conformation of SERCA obtained in the first crystal structure (Fig. 1, left). In the molecular dynamics study, a large-scale conformational change was observed in which the cytoplasmic head-piece closed dramatically both in the presence and absence of Ca2+, with a slightly more closed conformation (by 4 Å) observed in the presence of Ca. That study showed that this extra Ca-dependent closure was necessary and sufficient for SERCA to reach the precise geometrical arrangement necessary for activation of ATP hydrolysis [35].

**Table 1:** Quantitative FRET of 2-Color SERCA Constructs.

FRET Efficiency (%)				
	510	577	662	C-term
**Intact cells**	15.9±0.2	16.8±0.3	7.0±0.3	6.5±0.2
**+ Ca**	20.6±0.3	18.0±0.2	7.2±0.1	8.1±0.2
**+ TG**	6.6±0.4	13.7±0.3	9.4±0.2	9.5±0.3
**+ EGTA**	5.3±0.1	8.3±0.1	4.5±0.2	5.8±0.6
**Probe Separation Distance (Å)**				
	**510**	**577** *	**662** *	**C-term**
**Intact cells**	65.7±0.2	65.0±0.2	76.6±0.6	77.7±0.4
**+ Ca**	62.4±0.2	64.1±0.1	76.3±0.2	74.7±0.3
**+ TG**	77.5±0.8	67.7±0.3	72.6±0.3	72.5±0.5
**+ EGTA**	80.5±0.3	74.3±0.2	82.9±0.6	79.3±1.5

* Incomplete fluorophore maturation for 576 and 661 may yield underestimated distance.

### Potential Strategy for Drug Discovery

Of the constructs described here, the least responsive was 661, which showed low initial FRET efficiency and small changes with Ca or Tg. This is consistent with inefficient maturation of the YFP tag (**Fig. 6**C) that is inserted in the SERCA P-domain. Future studies might benefit from using alternative fluorescent protein tags or different P domain labeling positions in order to study the conformational dynamics of this part of SERCA. Other variants performed better, particularly 509, which showed large changes in FRET in response to Ca and Tg. Although the fluorescent protein fusion tags are large, our previous studies and present data indicate they are benign for function [14] (**Fig. 3** **,** **Fig. 4**) and can report conformational changes [16], [19] (**Fig. 7**). In addition, this method offers several advantages over conventional covalent labeling strategies. Being genetically encoded, fluorescent proteins may be directed to arbitrary labeling sites with fixed stoichiometry, and they are suitable for *in vivo* applications. In addition, the constructs are amenable to incorporation into a stable cell line [36], making this FRET assay readily scalable. We anticipate that 2-color SERCA will be useful for screening candidate small molecule modulators of SERCA function.
